# A pilot feasibility randomised clinical trial comparing dialkylcarbamoylchloride‐coated dressings versus standard care for the primary prevention of surgical site infection

**DOI:** 10.1111/iwj.13113

**Published:** 2019-03-14

**Authors:** Joshua P. Totty, Louise H. Hitchman, Paris L. Cai, Amy E. Harwood, Tom Wallace, Dan Carradice, George E. Smith, Ian C. Chetter

**Affiliations:** ^1^ Academic Vascular Surgical Unit Hull Royal Infirmary Hull UK

**Keywords:** dialkycarbamoylchloride, RCT, surgical site infection, wound dressings

## Abstract

A surgical site infection (SSI) may occur in up to 30% of procedures and results in significant morbidity and mortality. We aimed to assess the feasibility of conducting a randomised controlled trial (RCT) examining the use of dialkylcarbamoylchloride (DACC)‐impregnated dressings, which bind bacteria at the wound bed, in the prevention of SSI in primarily closed incisional wounds. This pilot RCT recruited patients undergoing clean or clean‐contaminated vascular surgery. Participants were randomised intraoperatively on a 1:1 basis to either a DACC‐coated dressing or a control dressing. Outcomes were divided into feasibility and clinical outcomes. The primary clinical outcome was SSI at 30 days (assessed using Centers for Disease Control criteria and Additional treatment, Serous discharge, Erythema, Purulent exudate, Separation of the deep tissues, Isolation of bacteria and duration of inpatient Stay scoring methods). This study recruited 144 patients in 12 months at a median rate of 10 per month. Eligibility was 73% and recruitment 60%. At 30 days, there was a 36.9% relative risk reduction in the DACC‐coated arm (16.22% versus 25.71%, odds ratio 0.559, *P* = 0.161). The number needed to treat was 11 patients. A large‐scale RCT is both achievable and desirable given the relative risk reduction shown in this study. Further work is needed to improve the study protocol and involve more centres in a full‐scale RCT.

## 1 INTRODUCTION

A surgical site infection (SSI) may complicate up to 40% of vascular surgical procedures, with those undergoing lower limb revascularisation and major limb amputations at the highest risk.^1^, ^2^, ^3^ In addition to significant mortality and morbidity, SSIs are known to increase length of stay by an average of 10 days and cost the National Health Service (NHS) an estimated £700 million per annum.^4^ The UK National Institute for health and Clinical Excellence and the World Health Organisation have publishing guidelines for the prevention of SSIs in recent years, which highlight the significant scale of the problem.^5^, ^6^, ^7^

Postoperative wound dressings provide a stable environment for a freshly incised wound, absorbing exudate and protecting the wound until epithelialisation can occur. Many dressings exist and have been the subject of wound research, yet no current systematic reviews have found evidence to suggest that any one dressing outperforms any other in the prevention of SSIs.^8^, ^9^

One technology not included in reviews to date is dialkylcarbamoylchloride (DACC), a fatty acid derivative that has been shown *in vitro* to bind to a number of pathogenic organisms, including *Pseudomonas aeruginosa* and methicillin‐resistant *Staphylococcus aureus* (MRSA).^10^, ^11^ This can be applied to dressings to irreversibly bind bacteria, thus removing them from the wound bed.^12^ Evidence in various types of surgery suggests that DACC‐coated dressings show promise in the prevention of SSIs.^13^ Robust randomised evidence is required to properly investigate the effectiveness of this technology.

The aim of this pilot study was to assess the methods and feasibility of conducting a definitive randomised clinical study of DACC‐coated dressings for the primary prevention of SSIs.

## 2 METHODS

A two‐arm, parallel‐group, pilot feasibility randomised controlled trial (RCT) was developed and conducted in a tertiary vascular surgery unit in the United Kingdom. The full protocol has previously been published by the same authors.^14^ Ethical approval was obtained (16/LO/2135), and study conduct was in accordance with the Declaration of Helsinki (1975).^15^ The study was prospectively registered with clinicaltrials.gov (NCT02992951).

Patients over 18 years old, undergoing clean or clean‐contaminated vascular surgery, and capable/willing to give informed consent were included. Those undergoing carotid endarterectomy, receiving antibiotics at the time of surgery, or with known allergies to trial dressings were excluded. All eligible patients provided informed written consent.

### 2.1 Outcomes

As a pilot/feasibility study, outcomes were divided into two distinct categories: *feasibility* outcomes and *clinical* outcomes.

#### 2.1.1 Feasibility outcomes

- Eligibility rates and reasons for non‐eligibility
- Participant recruitment rates and reasons for non‐recruitment
- Follow‐up and study retention rates and reasons for dropout/non‐attendance
- The suitability of the inclusion/exclusion criteria
- The suitability of outcome assessment measure(s)
- Fitness for purpose of follow‐up arrangements
- Rates of participant withdrawal from the trial.

### 2.2 Clinical outcomes

#### 2.2.1 Primary clinical outcome

The incidence of SSIs within 30 days of surgery, measured by an Additional treatment, Serous discharge, Erythema, Purulent exudate, Separation of deep tissues, Isolation of bacteria and duration of inpatient Stay (ASEPSIS) score ≥ 21^16^, ^17^ or according to the Centers of Disease Control (CDC) definition of SSIs.^18^, ^19^

#### 2.2.2 Secondary clinical outcomes

- The incidence of SSIs at 90 days for implant patients only
- Satisfactory healing—total ASEPSIS score ≤ 10 at 30 days post‐surgery for non‐implant surgery and implant patients
- Satisfactory healing—total ASEPSIS score ≤ 10 at 90 days post‐surgery for implant patients only

#### 2.2.3 Randomisation, blinding, and study procedures

Participants were randomised to postoperative wound dressing with either a DACC‐coated occlusive absorbent dressing (Leukomed Sorbact, BSN Medical, Hull, UK) or a non‐DACC‐coated occlusive absorbent dressing (OPSITE Post‐op, Smith & Nephew, Hull, UK) in a 1:1 ratio. Randomisation was performed in the theatre after wound closure to prevent performance bias using computer‐generated numbers in random permuted blocks via an online randomisation service (Sealed Envelope Ltd, London, UK). Randomisation was stratified for prosthetic implant/non‐implant, wound site (upper limb/lower limb/trunk), and diabetes (yes/no).

This was an open‐label trial as the differing appearances of the dressings prevented blinding. All patients received standardised care preoperatively, including surgical skin antisepsis, antibiotic prophylaxis for all patients undergoing procedures under general anaesthetic as per loco‐regional guidance, hair clipping, intra‐ and postoperative warming, and oxygen therapy. Initial dressings were applied in the operating theatre under sterile conditions. Where patients had more than one incision, all wounds were dressed according to dressing allocation.

#### 2.2.4 Follow‐up procedures

Follow‐up visits took place between postoperative days (POD) 5 and 7 and at POD 30 ± 3 days. Wounds were assessed using the ASEPSIS scale^16^, ^17^ by an investigator blinded to the allocated dressing. SF‐36 and the EQ‐5D questionnaires were completed. Telephone contact with non‐attenders and postal questionnaires were completed to avoid missing data. Further telephone contact was made at 3, 6, and 12 months postoperatively (±2 weeks) to capture wound problems occurring beyond POD 30.

### 2.3 Statistical analysis

Data were collected and entered into IBM SPSS (IBM SPSS Corporation, version 23; Rochester, New York), and a two‐sided *P*‐value of <0.05 was considered the level of significance where appropriate.

Clinical outcome data were analysed on an intention‐to‐treat (ITT) basis. The groups were compared using Pearson's *χ* ^2^ test or Fisher's exact tests for categorical data and *t*‐tests for continuous data. The primary outcome, presence or absence of SSI, was compared using *χ* ^2^ tests.

For the primary outcome, logistic regression analysis was undertaken with SSI as the dependant variable and randomisation group as an independent variable. The model was adjusted for confounding variables and surgical site. The regression model performance was assessed using the Hosmer and Lemeshow Test.

## 3 RESULTS

**Figure 1:**
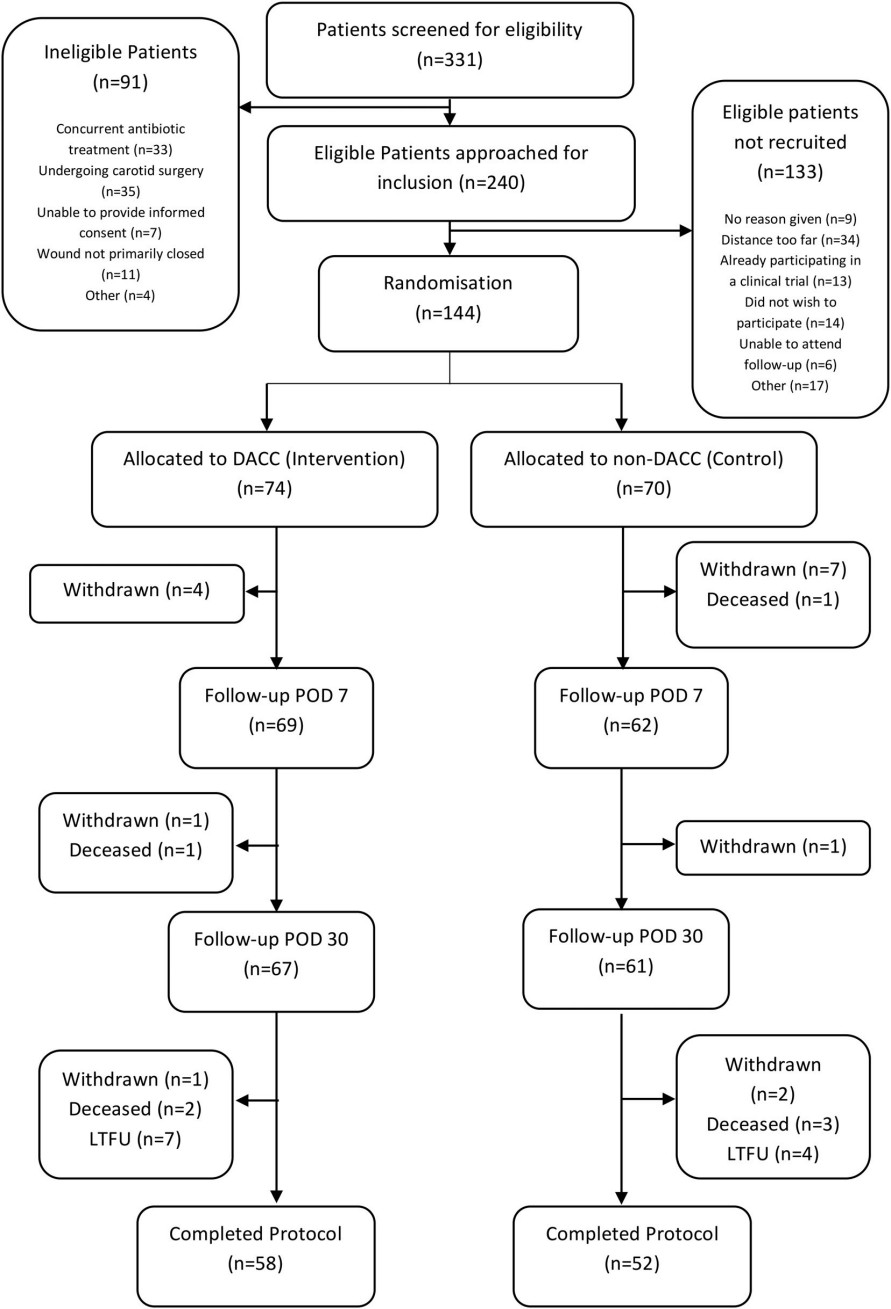
CONSORT diagram. DACC, dialkylcarbamoylchloride; POD, postoperative day; LTFU, lost to follow up

Recruitment commenced on the 19th January 2017 and was completed by 7th February 2018. Patient flow through the trial is shown in Figure 1. Tables 1 and 2 outline the baseline characteristics, procedures performed, and intraoperative procedures for each group.

**Table 1:** Baseline characteristics

	Non‐DACC coated (*n* = 70)	DACC coated(*n* = 74)
Male (*n*)	46	48
Female (*n*)	24	26
Age (yr)	62.36 (±12.31)	63.91 (±12.38)
BMI (kg/m^2^)	27.73 (±5.89)	27.65 (5.84)
Smoking status (*n*)		
Never	15	18
Ex	40	35
Current	15	21
Diabetes mellitus (*n*)		
None	47	54
Diet controlled	3	1
Tablet controlled	9	9
Insulin dependent	11	10
CVA (*n*)	8	8
Hypertension (*n*)		
Uncontrolled	3	2
One agent	25	14
Two agents	10	22
Three or more agents	11	11
Cardiac disease (*n*)	28	30
PVD (*n*)	35	37
Respiratory disease (*n*)	14	16
Renal impairment (*n*)	16	19
Baseline creatinine (μmol/L)	160 (±182)	152 (±198)
Procedure performed (*n*)		
Open abdominal	14	12
Lower limb arterial	29	28
Open varicose vein	6	8
Major limb amputation	7	9
Renal dialysis access	5	8
Other	9	9

BMI, body mass index; CVA, cerebrovascular accident; DACC, dialkylcarbamoylchloride; GI, gastrointestinal disease; PVD, peripheral vascular disease.

**Table 2:** Intraoperative details

	Non‐DACC coated (*n* = 70)	DACC coated (*n* = 74)
ASA grade		
Not recorded	10	12
1	6	6
2	11	17
3	39	34
4	4	5
5	0	0
Surgeon grade		
Consultant	39	45
Senior StR	25	20
Junior StR	5	9
Core trainee	1	0
Other	0	0
Closure method		
Continuous suture	1	3
Interrupted suture	3	10
Subcuticular suture	61	58
Skin clips	3	3
Drain placed	7	5

ASA, American Society of Anaesthesiologists; StR, specialty training registrar.

## 4 FEASIBILITY OUTCOMES

### 4.1 Eligibility rate

A total of 331 patients were screened for eligibility for inclusion in the study. Of these 331, 240 patients were eligible (73%). Common reasons for ineligibility included patients undergoing carotid surgery (35 of 91 ineligible patients, 39%) or concurrent antibiotic therapy at the time of the index procedure (33 of 91 ineligible patients, 37%).

### 4.2 Recruitment rate

Of the 240 eligible patients, 144 (60%) agreed to participate.

A variety of reasons for eligible patients not being recruited were encountered; most commonly, patients did not wish, or were not able, to attend follow‐up visits (40 of 96 patients, 41%).

Median recruitment rate was 10 patients per month (IQR 8.25‐12.75).

### 4.3 Study retention, dropouts, and reasons for withdrawal

Four patients were recruited but subsequently not randomised; one patient decided not to undergo surgery, one patient withdrew their consent to participate in the trial prior to randomisation, one patient was not randomised because of an error in theatres, and one patient's procedure was cancelled.

Sixteen patients actively withdrew from the trial during the study period. The most common reason for withdrawal was an inability or an unwillingness to attend study follow‐up visits (8 of 16 withdrawals).

Seven patients died in follow up, unrelated to study outcomes or interventions, with two deaths within 30 days of the procedure (pneumonia and myocardial infarction). Eleven patients attended no follow‐up visits and returned no questionnaires. This amounts to a combined dropout rate of 23.6% (34 patients of 144).

### 4.4 Follow‐up rates and reasons for non‐attendance

Of 131 possible POD 5 to 7 visits (not including patients who had died or withdrawn from the trial), 95 were completed (73%). Of 128 possible POD 30 ± 3 visits, 81 were completed (63%). Data on SSI within 30 days were available for 119 participants (82.6%). Three patients withdrew from the trial after experiencing an SSI; their data were included in the final primary clinical outcome analysis.

### 4.5 Suitability of the trial interventions

Sixteen protocol deviations related to trial interventions were recorded. Nine patients were found have non‐protocol dressings in situ, with no reason given for the change. Two patients had non‐protocol dressings as their wounds required the placement of more absorbent dressings. One patient in the DACC arm experienced a desquamating allergic reaction to a perioperative antibiotic, necessitating a non‐adhesive dressing to be used.

After experiencing SSIs, two patients in the control arm had inadine applied on the wound; one patient in the control arm had wound dressing with absorbent pads, followed by silver nitrate dressings, and one patient in the DACC arm had negative pressure in situ.

## 5 CLINICAL OUTCOMES

### 5.1 SSI within 30 days of surgery

Fewer patients in the DACC‐coated group had an SSI at 30 days than the control group (12/74 (16%) and 18/70 (26%) respectively). The difference was non‐significant (*P* = 0.161, Pearson's *χ* ^2^ test). This represents an estimated absolute risk reduction of 10%, a relative risk reduction of 37%, and a number needed to treat of 10.5 patients. The crude odds ratio (OR) was 0.559 [95% CI: 0.247, 1.267]. There were no significant differences found between groups in each surgery subtype.

Presence of diabetes, peripheral vascular disease (PVD), and the type of surgery performed were the only factors that had a statistically significant effect on rates of SSI. Of 30 patients who experienced an SSI, 14 had diabetes, compared with 29 of 114 who did not experience an SSI (*P* = 0.024). In the SSI group, 20 patients had PVD (66.7%) versus 52 in the no SSI group (46.0%), a statistically significant difference (*P* = 0.044). More patients who experienced SSI had lower limb arterial surgery or major limb amputations than those who did not experience SSI (56.7% and 20.0% versus 35.1% and 8.8% respectively, *P* = 0.029). Current or ex‐smokers were seemingly less likely to experience infection: 14 patients of the 30 with an SSI were current or ex‐smokers (60%), in comparison with 93 of the 114 patients without an SSI (81.6%) (*P* = 0.012). With regard to intraoperative factors, the only significant difference between the two groups was that more patients in the SSI group had the placement of a drain (26.7%) than in the no SSI group (3.5%) (*P* < 0.001). With regard to antibiotic use, in those who experienced SSI in the control group, 10 patients required oral antibiotics within 30 days of their procedure, and 7 required intravenous (IV) antibiotics. In the DACC‐coated group, seven required oral antibiotics and five IV.

### 5.2 Controlling for confounding variables

A binomial logistic regression analysis was undertaken to ascertain the effects of various factors, including dressing group allocation, on the likelihood of experiencing SSI. Gender, age, BMI, ASA grade 3 or higher, presence or absence of PVD, diabetes, smoking status, procedure performed, and randomisation group were included in the model. The model explained 44.2% (Nagelkerke *R* ^2^) of the variance in SSI and correctly classified 86.7% of cases. Table 3 summarises the results of the logistic regression analysis.

**Table 3:** Binomial logistic regression analysis of identified variables associated with an increase in incidence of SSI

Variable	Wald	df	Sig	OR	95% CI	
					Lower	Upper
Gender (male)	6.187	1	0.013*	5.850	1.454	23.530
Age	0.167	1	0.682	1.010	0.963	1.060
BMI	4.765	1	0.029*	1.109	1.011	1.216
Presence of PVD	1.030	1	0.310	2.107	0.500	8.883
Current or previous smoker	7.894	1	0.005*	0.144	0.037	0.556
Presence of diabetes	0.049	1	0.824	1.131	0.381	3.360
Randomisation group	2.584	1	0.108	0.423	0.148	1.208
ASA grade ≥ 3	0.722	1	0.396	1.742	0.484	6.273
Surgical procedure performed^a^	6.105	1	0.013*	7.321	1.509	35.515
Placement of a Drain	6.166	1	0.013*	8.560	1.572	46.610

a Surgical procedure performed is dichotomised into infra‐inguinal surgery and other vascular surgeries.

* denotes statistical significance. ASA, American Society of Anaesthesiologists; CI, confidence interval; df, degrees of freedom; OR, odds ratio; Sig, significance.

### 5.3 Satisfactory healing within 30 days of procedure

More patients achieved satisfactory healing within the first 30 days in the DACC‐coated group than the control group (62.3% versus 50.0%). The difference was non‐significant (*P* = 0.236, Pearson's *χ* ^2^ test).

### 5.4 SSI at 30 and 90 days for the implant subgroup

A total of 51 patients (35.4% of total) received a prosthetic implant (26 patients in the DACC‐coated arm, 25 in the control arm). In this group, all observed incidences of SSI occurred between procedure and POD 30; no new infections occurred between POD 30 and POD 90 time points. At 30 days, there was a non‐significant difference in infection rates between the two randomisation groups, with a 24.0% infection rate in the control group, falling to 7.7% in the intervention group (*P* = 0.109, Pearson's *χ* ^2^ test).

## 6 DISCUSSION

This pilot study has provided invaluable information about the feasibility of conducting a large‐scale RCT investigating the effectiveness of DACC‐coated postoperative dressings for the prevention of SSIs. Eligibility, retention, and recruitment rates were consistent with other studies in this area^20^, ^21^ but could be further optimised as part of a subsequent larger study to maximise efficiency and costs.

Despite studies of a similar scale reporting only 10% of screened subjects recruited,^22^ 43.5% of the patients screened in this trial were subsequently randomised. Patients were recruited at a median rate of 10 per month. The consistent recruitment rate suggests it would be feasible to recruit to a large‐scale trial and provides an estimate of the number of sites to be identified at the outset.

Patients not wishing to take part in research or patients who were unable or unwilling to re‐attend hospital for additional clinical visits made up over half of eligible patients who were subsequently not recruited. Travel problems, and additional appointments or costs to patients, are established barriers to participation in clinical research.^23^, ^24^, ^25^, ^26^ Follow‐up procedures for a larger trial would need to be augmented to facilitate recruitment as over 40% of eligible patients not recruited cited such problems as a reason for not entering the trial.

Amending follow up might also impact the 20% attrition rate seen in this pilot study. Attrition of this level may compromise study validity^27^ or alter the significance of results depending on how missing data are handled.^28^ Telephone or remote follow up is an option as only 63% of participants attended a clinical review at POD 30 ± 3, although primary outcome data were available for 82.6% of participants using notes review and telephone contact. More contemporary technology might be used during a clinical trial^29^ to improve communication with patients and avoid in‐person attendances for follow‐up data collection.^30^, ^31^ Prior examples of remote follow up are already being reported in clinical trials,^32^, ^33^, ^34^, ^35^ and in routine clinical practice.^36^, ^37^, ^38^, ^39^ Specific work aimed at identifying SSIs using remote technology is already underway.^40^, ^41^ Combining this growing area of interest with established methods of improving follow up, such as offering incentives, may improve study recruitment and retention.

The ASEPSIS scoring system, used to identify SSIs in post‐surgical wounds, has been shown to be reliable and related to patient outcomes.^17^, ^42^, ^43^ However, the ASEPSIS score remains only one of a number of definitions of SSI, and our study combined the use of ASEPSIS and CDC definitions of SSI.^18^, ^19^ To achieve data that accurately reflect true SSI rates, a combination of assessment methods, involving searching existing computerised data for GP attendances or antibiotic prescriptions, telephone consultations, validated questionnaire use, and targeted clinical review, should be used.^41^, ^44^, ^45^ Several study groups are currently designing, or validating, novel methods for the follow up of patients post‐discharge, which aim to increase the capture rate of data on SSI in both research trials and clinical practice,^41^, ^46^ and incorporating one or more of these tools may benefit any RCT arising as a result of this pilot study.

The effect size seen in this pilot study was a reduction in SSI at 30 days from 26% to 16%, an ARR of 10%, and an RRR of 36.9%. In a full‐scale, two‐arm RCT based on this study design, for 90% power and 5% significance, a total of 772 participants would be required (Calculated using *OpenEpi*, Open Source Epidemiologic Statistics for Public Health^47^). With no adjustment to reduce attrition from near 20%, a total sample size of 925 might be required. At the median recruitment rate of 10 participants per centre per month, completing study recruitment in 18 months would require approximately five centres to take part in the trial.

There are a number of clinical limitations to this study and, therefore, any potential future studies. First, the wide range of surgical procedures performed introduces a level of heterogeneity into the study that may impact the results. Although randomisation may go some way to limiting the effects of this (by ensuring similar numbers of procedure types in each arm of the trial), future studies could focus on a smaller group of procedures, such as those involving an infra‐inguinal incision. This may limit any bias introduced through this heterogeneity. Second, this study was conducted as an open‐label study, although blinded outcome assessors were used. Future studies could maximise the use of blinded outcome assessors, and work is underway to validate new tools that can be used in such circumstances.^35^, ^40^

## 7 CONCLUSIONS

As a pilot feasibility study, this study has shown that a large‐scale RCT is achievable and desirable. Amendments in the follow‐up protocol can be made to reduce the burden to patients and improve the levels of data return. Dressings used in this study are tolerable and showed no adverse reactions. Furthermore, with an estimated relative risk reduction of over 35%, robust evidence to support the clinical and cost‐effectiveness of DACC‐coated dressings could strongly influence current surgical practice.

## CONFLICT OF INTERESTS

The authors declare that BSN Medical (Hull) provided the intervention dressing for this study. The company had no input into trial design, conduct, analysis, or dissemination.
